# Synovial sarcoma of the supraclavicular region causing a hemorrhagic shock

**DOI:** 10.1002/ccr3.2270

**Published:** 2019-09-24

**Authors:** Sara Halily, Amine Ballage, Hamida Ardhaoui, Sami Rouadi, Redallah Abada, Mohamed Roubal, Mohamed Mahtar, Hasna El Khiraoui, Salwa Belhaj, Farida Marnissi

**Affiliations:** ^1^ Otorhinolaryngology Department Casablanca Teaching Hospital Casablanca Morocco; ^2^ Pathology Department Casablanca Teaching Hospital Casablanca Morocco

**Keywords:** hemorrhagic shock, supraclavicular region, synovial sarcoma

## Abstract

A cystic mass that rapidly increases in volume in a dangerous anatomical region, notably the supraclavicular region, which contains important neurovascular elements, must be considered at high risk. A vascular study is mandatory before biopsy. Neoadjuvant chemotherapy can be a treatment option in high‐risk synovial sarcoma.

## Graphical abstract

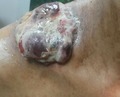

## 1 INTRODUCTION

Synovial sarcomas (SS) are rare malignant soft tissue tumors, which account for approximately 5%‐10% of all soft tissue sarcomas.^1^ They are termed SS because of their histologic resemblance to the synovium, but they rarely involve a synovial structure and are thought to arise from pluripotential mesenchymal cells.^2^

It most commonly affects young adults of the second to fourth decade and usually occurs in para‐articular locations of the extremities, although it may be found in areas unrelated to synovial tissues.^3^ Since the first case of head and neck SS reported by Jernstrom in 1954, only 3%‐5% of all cases were found in the head and neck region. In this region, the hypopharynx is the most common site.^4^

## 2 CASE PRESENTATION

We report a case of a 14‐year‐old girl presenting in the otorhinolaryngology department of Casablanca teaching hospital with a 4‐month history of a painless, progressively enlarging mass in the right side of the neck. There were no symptoms of fever, weight loss, dysphagia, or dyspnea.

**Figure 1:**
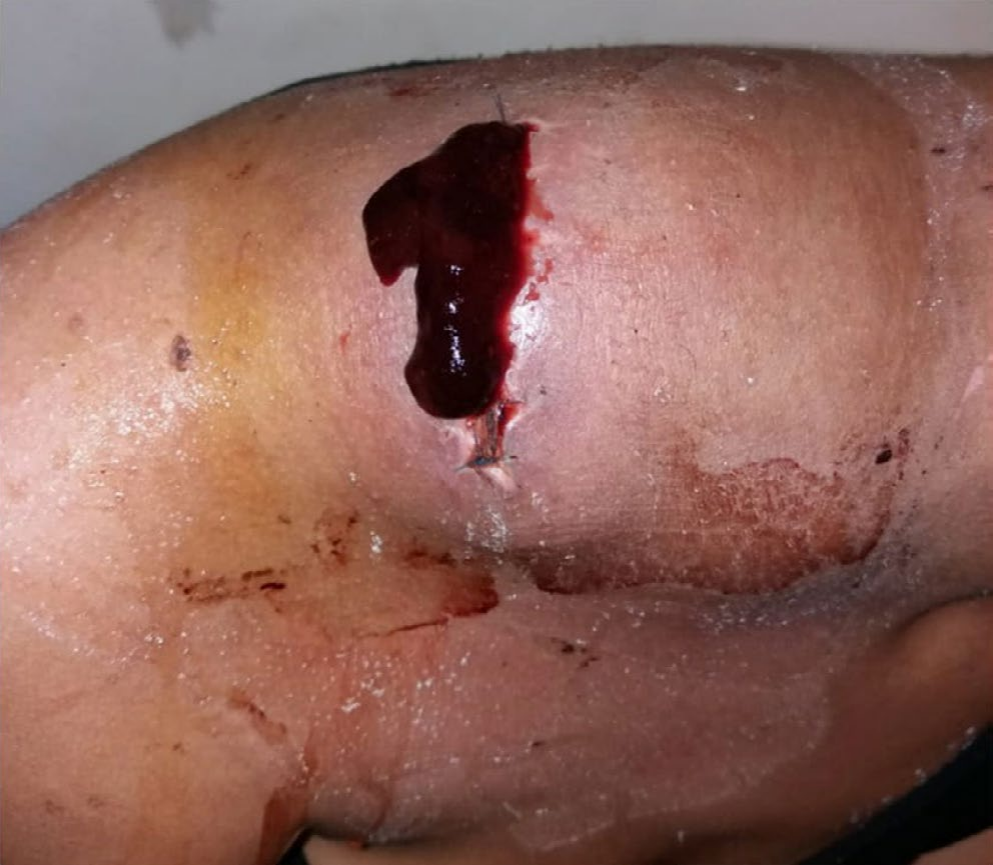
Cystic mass of the right supraclavicular region with necrotic viscous liquid leaking from the incision after the biopsy

Physical examination revealed a large (9 × 7 cm), elastic, nontender, and nonpulsatile mass located in the right supraclavicular region of the neck (Figure 1). On laryngoscopic examination, the pharyngeal and laryngeal mucosa was intact and bilateral vocal cords were normally mobile.

**Figure 2:**
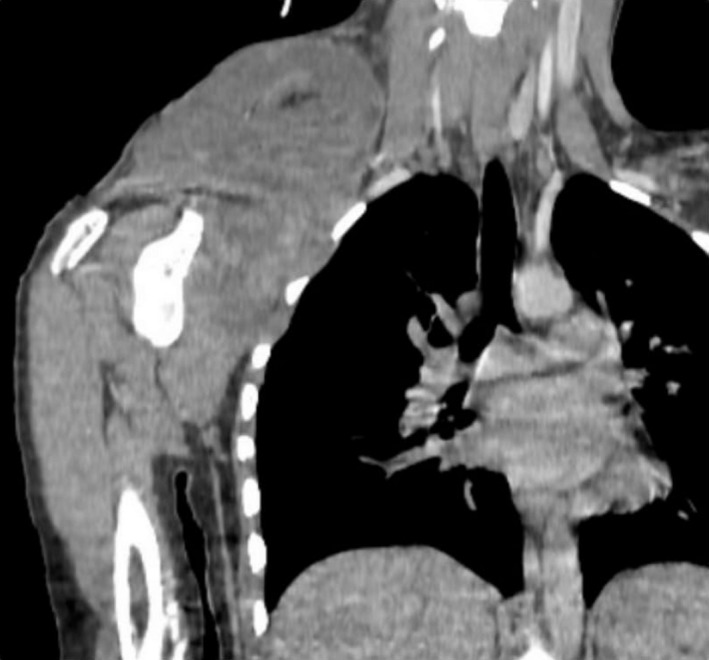
CT scan showing a voluminous tumor of the right supraclavicular region extending over 9 cm to the 5th intercostal space

CT scan showed a voluminous solido‐cystic tumor of the right supraclavicular region with predominantly fluid component (density 23 HU) and spontaneously hyperdense zones (54 HU). This mass enhances slightly after injection of contrast medium in a heterogeneous manner. It measures 9.4 × 7.7 cm and extends over 9 cm. The tumor arrives down to the 5th right intercostal space. At the top, it comes into contact with the lateral muscles of the neck. We note a laminar appearance of the axillary vascular pedicle and infiltration of adjacent fat. In addition to bilateral cervical and right axillary adenopathies, some of which were necrotic. The right axillary adenopathy is the largest measuring 14.8 × 8.5 mm (Figure 2).

Contrast‐enhanced MRI was taken into consideration to assess in a more accurate way the nature and vascularization of the tumor; but since it was not available in a short term, it would have significantly delayed treatment.

A neck exploration was performed under general anesthesia. A dark viscous, necrotic liquid came out after the incision was made, followed by a persistent bleeding originating from the tumor. This caused hemodynamic instability requiring transfusion of red blood cells, platelets, and fresh frozen plasma. Several biopsies were taken. The patient stayed four days in the intensive care unit without further complications.

**Figure 3:**
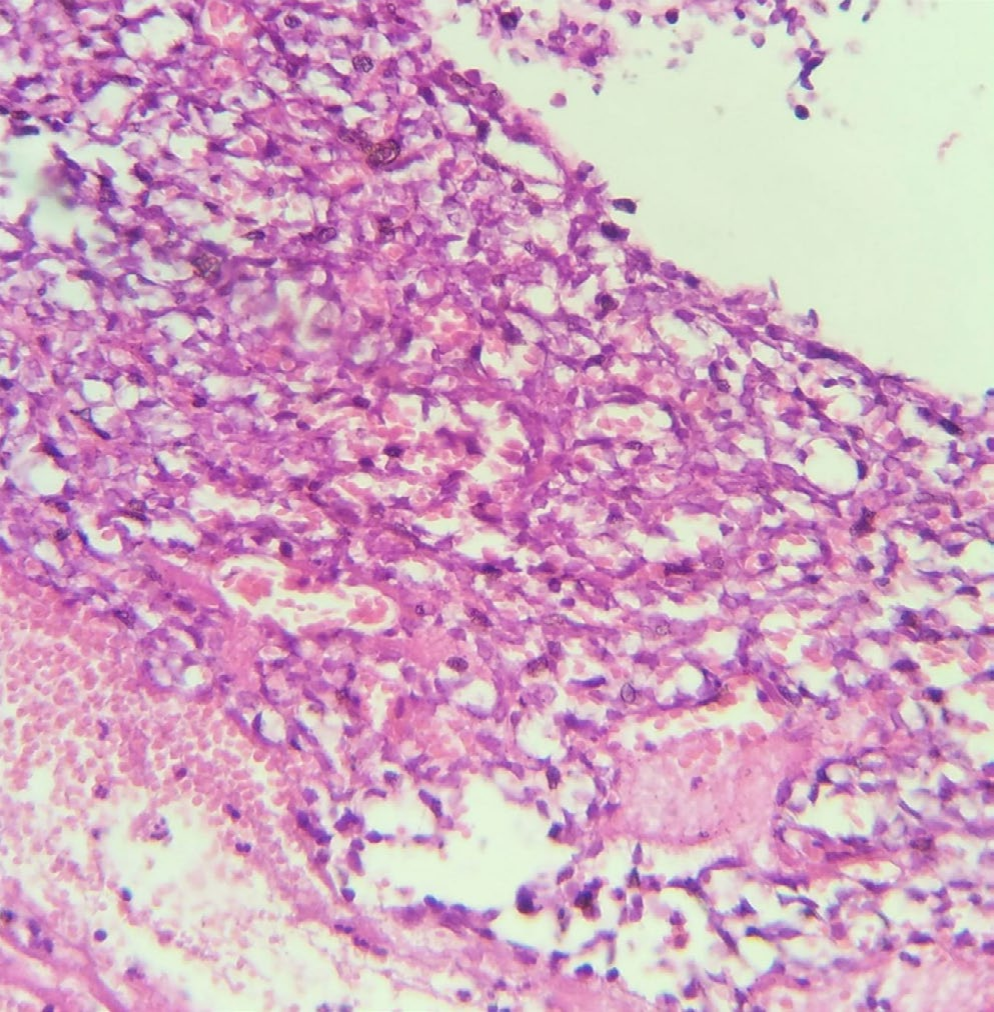
Biopsy specimen (hematoxylin‐eosin ×40) demonstrating monomorphic cells

**Figure 4:**
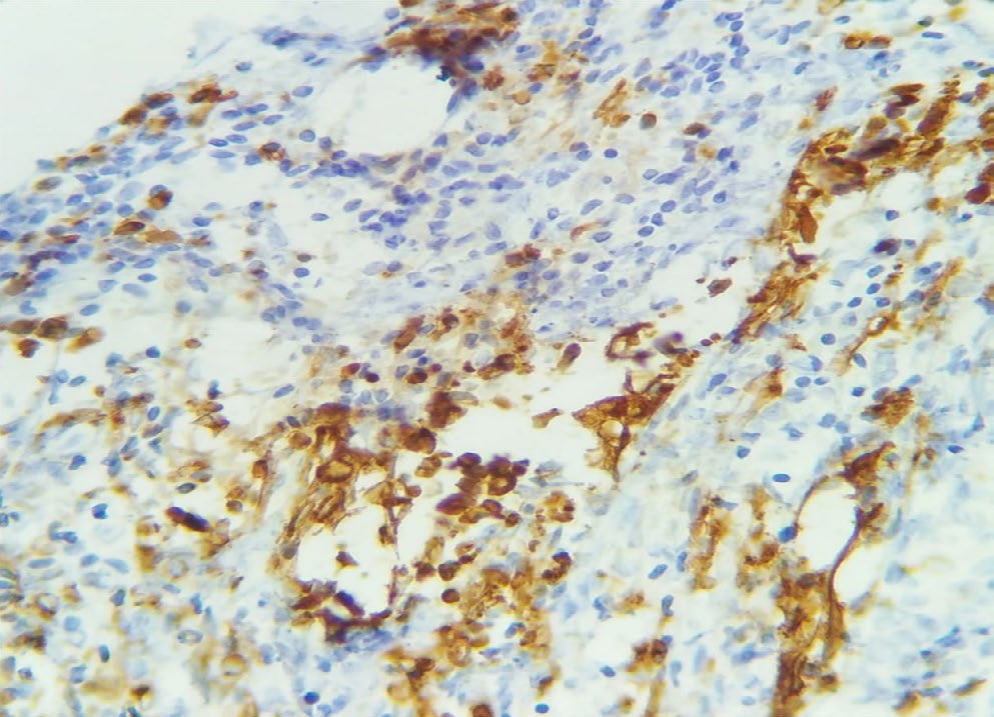
Immunohistochemical staining expressing CK7 (×40)

**Figure 5:**
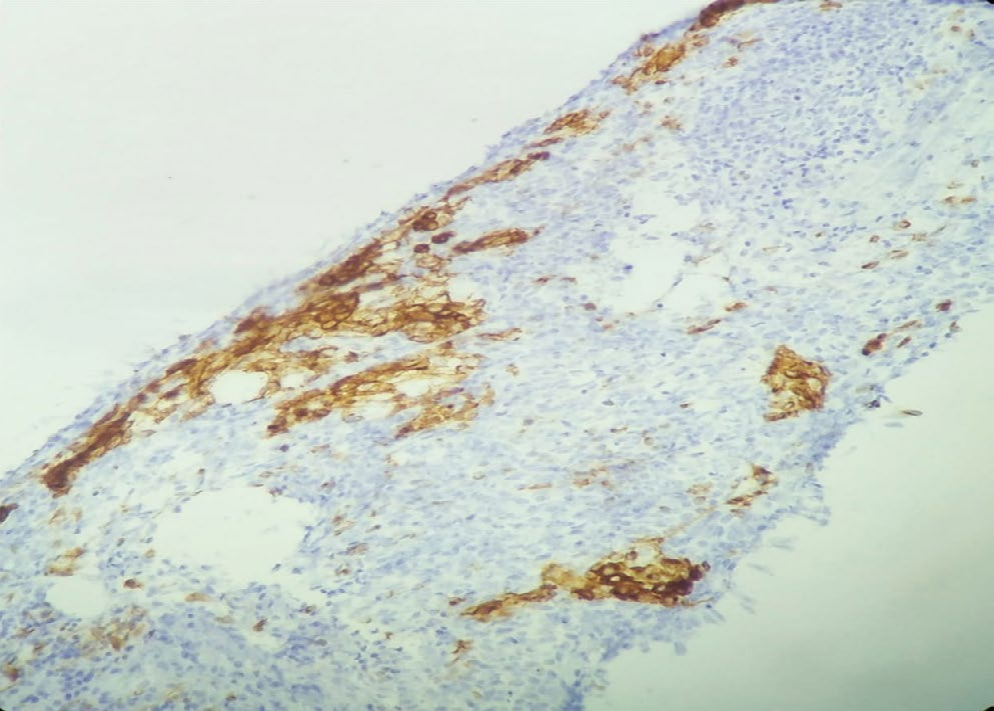
Immunohistochemical staining expressing CKAE1/AE3 (×20)

The histological examination of the surgical specimen demonstrated a cystic tumor composed of fascicles of spindle cells with a very rich vascularization. The tumor cells were monomorphic with poorly defined cytoplasm and oval to elongated nuclei with homogeneous chromatin (Figure 3). Mitoses were frequent (four per high power field). The immunohistochemical staining showed that tumor cells express AE1/AE3 cytokeratins, CK7, and epithelial membrane antigen (EMA) (Figures 4 and 5). Desmin, smooth muscle actin, S100, and CD31 and CD34 were all negative.

On the basis of the histological and immunohistochemical findings, the pathological diagnosis of a monophasic synovial sarcoma in its cystic variant grade II of the FNCLCC was made by a senior pathologist.

The multidisciplinary meeting decided to start with neoadjuvant chemotherapy to downstage the disease, improve local surgical control, and reduce wide excision morbidity. Surgery was not chosen as first option because the patient was considered high risk for two main reasons: large tumor size superior and dangerous site of presentation (anatomical relation to the subclavian and axillary vessels).

After five courses of chemotherapy, an angio‐MRI was performed. It showed 42% reduction in tumor size. The tumor came into contact with the subclavicular vessels without invading them and passed above the axilla. No feeder vessel was identified; therefore, embolization was not performed.

**Figure 6:**
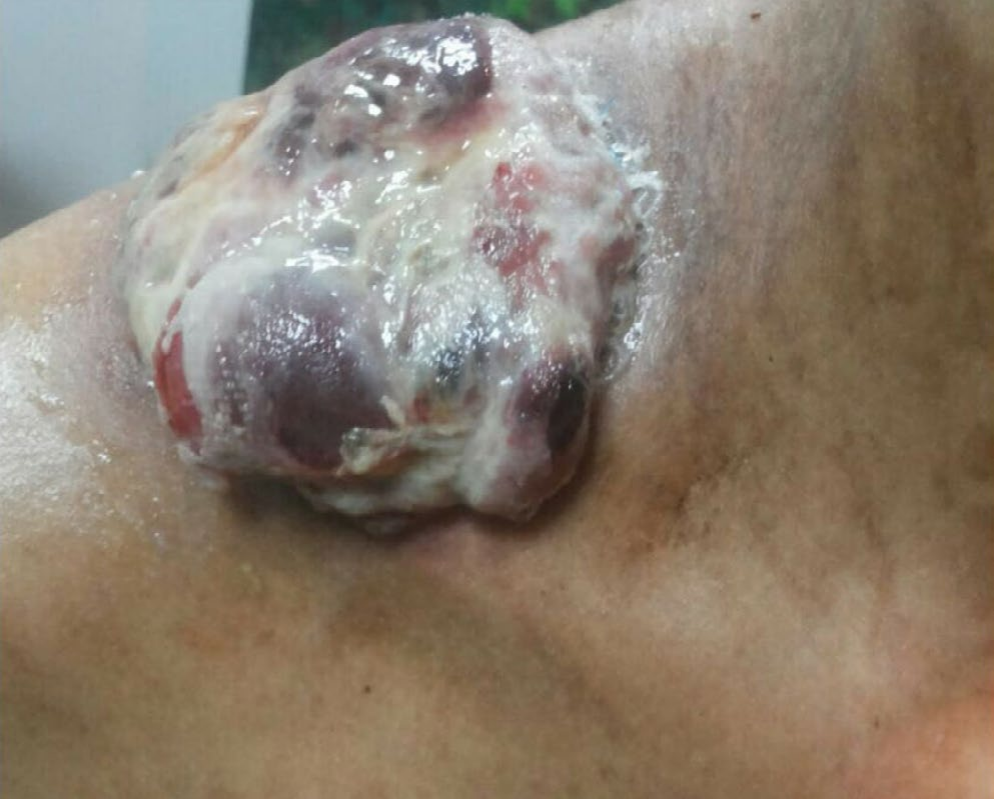
Evolution of the macroscopic aspect of the tumor after three courses of chemotherapy

**Figure 7:**
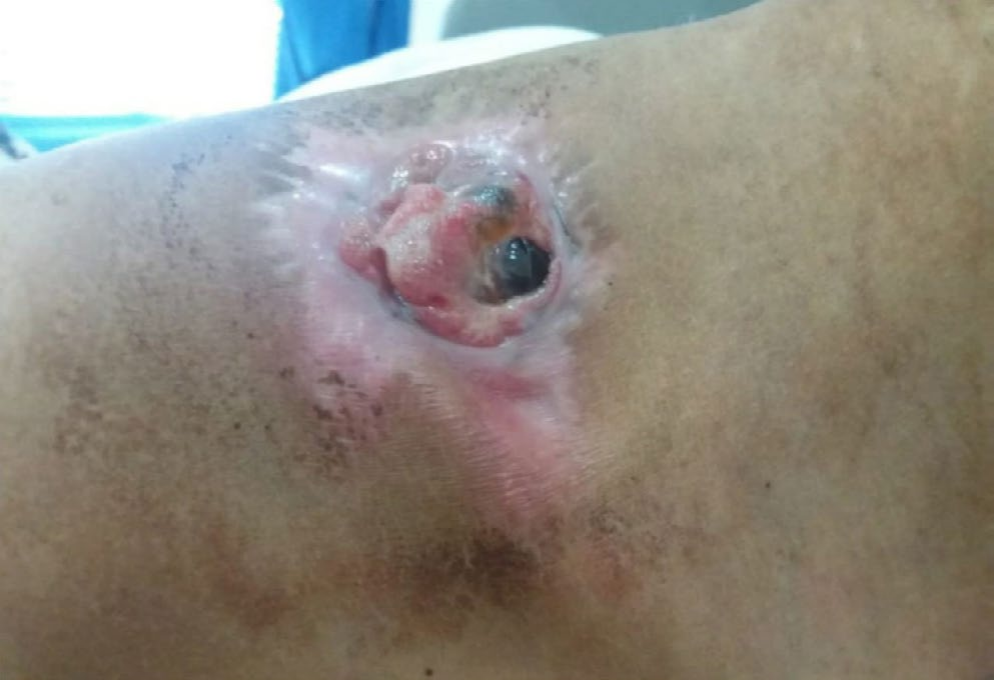
Tumor size after five courses of chemotherapy

The patient was staged II A (T1 N0 M0, Grade 2) (Figures 6 and 7). The surgery was performed by head and neck and thoracic surgeons without complications.

Adjuvant radiation therapy to the neck was administered. The patient was disease free until her six‐month follow‐up with no evidence of local recurrence or distant metastasis.

## 3 DISCUSSION

Synovial sarcoma (SS) is a mesenchymal malignancy that is termed SS since histological appearance is similar to that of the synovium. However, SS rarely exhibits a synovial structure and is considered to originate from pluripotent mesenchymal cells.^5^ SS frequently affects the lower extremities of adults from the second to fourth decades of life.^4^ It accounts for less than 10% of all tissue sarcoma of the head and neck where the most commonly involved sites are the hypopharynx and the parapharyngeal space.^4^, ^6^, ^7^

The diagnosis of SS is made on the basis of histopathological appearance, immunochemistry, and cytogenetic analysis which proved valuable in confirming morphological diagnosis.^5^

Interestingly, SS shows a considerable morphologic heterogeneity in which distinct histological subtypes can be distinguished: monophasic, containing spindle‐shaped mesenchymal cells; biphasic, containing both mesenchymal and well‐developed glandular epithelial cells; and the poorly differentiated subtype.^8^

SS harbors a highly specific, usually balanced and reciprocal t(X;18)(p11.2;q11.2) translocation, in which the SS18 (formerly SYT) gene (at 18q11) fuses with SSX genes: SSX1, SSX2, or rarely SSX4 (all at Xp11), leading to the generation of SS18‐SSX fusion oncogenes.^9^ This translocation can be easily detected using reverse transcription‐polymerase chain reaction (RT‐PCR) or fluorescence in situ hybridization (FISH).^4^

Our senior pathologist did not use PCR because the histological and immunochemistry findings were in favor of the diagnosis.

Optimal treatment of head and neck synovial sarcoma (HNSS) is controversial and is likely to vary according to the size of the tumor on presentation. The most common treatment of these tumors is surgery, followed by radiotherapy.

In a study of 167 HNSS, Mallen‐St et al reported that 89.8% of patients had surgery and 64.77% had radiotherapy, as radiotherapy improved disease‐specific survival (*P* = 0.003).^6^ Neoadjuvant chemotherapy should be given only in the presence of poor prognostic factors such as large tumor size (>5cm) or unfavorable site of presentation which was the case for our patient with tumor measuring 9 cm, localized in a dangerous region and extending to the axilla.^10^ Randomized trials have failed to show a survival benefit for adjuvant chemotherapy in adult SS.^8^ Therefore, it is used more frequently for large tumors, extensive or recurrent disease, and high‐risk sites of presentation, such as the skull base or paraspinal neck.^6^, ^11^

There are reports of successful immunotherapeutic treatments in several SS subtypes, with the best results being observed in those treated with interferon‐alpha combined with SYT‐SSX‐derived peptide vaccines and an adjuvant.^12^, ^13^

HNSS has a serious prognosis as several studies have reported a 5‐year overall survival (OS) rates ranging from 40% to 70% with a recurrence rate of 40%.^5^, ^6^, ^14^, ^15^

In a review of 44 patients with HNSS, primary tumor size was a predictor of progression (*P* = 0.008).^7^ Larger tumors have been found to be associated with poorer OS and progression‐free survival (PFS). Another study of 167 HNSS demonstrated that the only significant independent determinants of survival include the size of the tumor (>5cm) and the stage of presentation.^6^ In addition, Wushou et al reported that tumor size larger than 5 cm was the only independent adverse prognostic factor for determining OS in a meta‐analysis of 93 patients, as it has a higher risk of local recurrence, distant metastasis, and mortality than those with tumors ≤5cm in diameter.^5^

Histologic subtype has not been known to influence survival in HNSS patient with no difference seen with PFS or OS based on subtype. Moreover, age has never been explicitly identified as a prognostic indicator for SS.^6^, ^7^, ^10^, ^14^

The hyper vascularized cystic variant of the tumor and its localization in a dangerous anatomical region in contact with the subclavian vessels and the axillary plexus were the main limitations to surgery.

## 4 CONCLUSION

Despite its rarity, SS should be considered in the differential diagnosis of cystic lesions in the head and neck. The optimal treatment needs to be more clearly elucidated. We cannot draw conclusions on the efficacy of treatment modalities we adopted on the long term as the follow‐up was short. The literature lacks randomized studies that evaluate the efficacy of neoadjuvant chemotherapy in head and neck synovial sarcoma. However, other reports showed effectiveness of neoadjuvant chemotherapy in locally advanced high‐risk synovial sarcoma of the trunk and extremities.^16^, ^17^ The recent observed activity of immunotherapy, particularly targeting NY‐ESO‐1, trabectedin, and a variety of angiogenesis inhibitors deserve further exploration.

## CONFLICT OF INTEREST

The authors have nothing to disclose.

## AUTHOR CONTRIBUTION

SH: involved in data acquisition and manuscript writing; AB and HA: performed manuscript preparation and review; SR: is the main surgeon; RA, MR, and MM: analyzed and interpreted the data; HK, SB, and FM: involved in pathology diagnosis.
